# Laparoscopic transcystic stenting with postoperative ERCP for the treatment of common bile duct stones: a safe alternative to intraoperative rendezvous ERCP—Data from the Swedish registry for gallstone surgery and ERCP (GallRiks)

**DOI:** 10.1007/s00464-026-12565-3

**Published:** 2026-01-21

**Authors:** Sara Johansson, Camilla Runfors, Gabriel Sandblom, Björn Lindkvist, Anders Thorell, Marcus Reuterwall Hansson

**Affiliations:** 1 Department of Surgery & Anesthesiology, Ersta Hospital, Stockholm, Sweden; 2 Department of Clinical Sciences, Danderyds Hospital, Karolinska Institutet, Stockholm, Sweden; 3 Department of Surgery, Södersjukhuset, Stockholm, Sweden; 4 Department of Clinical Science and Education, Södersjukhuset, Stockholm, Sweden; 5 Department of Internal Medicine, Sahlgrenska University Hospital, Gothenburg, Sweden

**Keywords:** Transcystic stent, Rendezvous ERCP, Common bile duct stones, Intraoperative cholangiography, Cholecystectomy, Fanelli

## Abstract

**Background:**

Common bile duct stones (CBDS) are a common finding during laparoscopic cholecystectomy, yet the optimal treatment strategy remains under debate. In Sweden, CBDS are usually managed by intraoperative endoscopic retrograde cholangiopancreatography using the rendezvous technique (RV–ERCP). An alternative treatment approach is laparoscopic transcystic placement of an endobiliary stent with postoperative ERCP (TCStent–ERCP). The aim of this study was to compare these two treatment strategies in terms of complication rates.

**Method:**

Data were extracted from the Swedish Registry for Gallstone surgery and ERCP (GallRiks) from three different hospitals between 2010 and 2023. All cholecystectomies in which CBDS were detected intraoperatively and ERCP was carried out, either during the same session or later, were collected. Procedures involving TCStent–ERCP or RV–ERCP were identified. Complications within 30 days related to the cholecystectomy as well as to the ERCP were compared between the two groups.

**Results:**

In total, 929 patients were included. The overall complication rates were 21/183 (11%) in the TCStent–ERCP group and 140/746 (19%) in the RV–ERCP group. In adjusted multivariable regression analysis, fewer overall complications were found using the TCStent–ERCP strategy compared with RV–ERCP (OR 0.60, 95% CI 0.35–0.97). However, no statistically significant differences were observed between the groups for surgical complications or post-procedural pancreatitis.

**Conclusion:**

Transcystic stenting with postoperative ERCP appears to be a safe alternative to intraoperative rendezvous ERCP for the management of CBDS encountered during intraoperative cholangiography. This might be beneficial in surgical units where resources required for unplanned ERCP are limited or lacking.

Common bile duct stones (CBDS) encountered during cholecystectomy represent a well-recognized clinical dilemma. CBDS are the leading cause of acute pancreatitis, a condition which carries a substantial risk of severe complications and even mortality [1, 2]. The optimal management of CBDS in patients with gallstone disease remains undefined, with treatment approaches varying across countries. In Sweden, where intraoperative cholangiography (IOC) is performed routinely, detection of CBDS have been reported in the range of 9 and 21% during elective and acute cholecystectomy, respectively [3]. The European guidelines recommend bile duct clearance in all patients with CBDS, symptomatic or not [4].

ERCP is a widely adopted technique for managing CBDS, but the procedure itself may result in serious complications, in particular post-ERCP pancreatitis (PEP). Intraoperative rendezvous ERCP (RV–ERCP) using an antegrade guidewire inserted through the cystic duct facilitates biliary cannulation and has been shown to reduce the risk of PEP [5]. RV–ERCP is the recommended standard of care in Sweden for the management of CBDS when the gallbladder is in situ. CBDS encountered by IOC are treated with intraoperative ERCP in 59% of acute and 76% of elective procedures in Sweden [6]. A relatively large number of Swedish surgeons are qualified to perform ERCP, whereas choledochotomy or transcystic stone extraction by choledochoscopy are rarely conducted, mainly due to traditional and organizational reasons. However, not all surgical units performing cholecystectomies have readily access to RV–ERCP, and therefore, many CBDS detected during cholecystectomy are not managed by RV–ERCP. In such situations, other strategies are called for. Placement of a transcystic endobiliary stent (Fig. 1) followed by postoperative ERCP (TCStent–ERCP) is one such alternative.

**Fig. 1:**
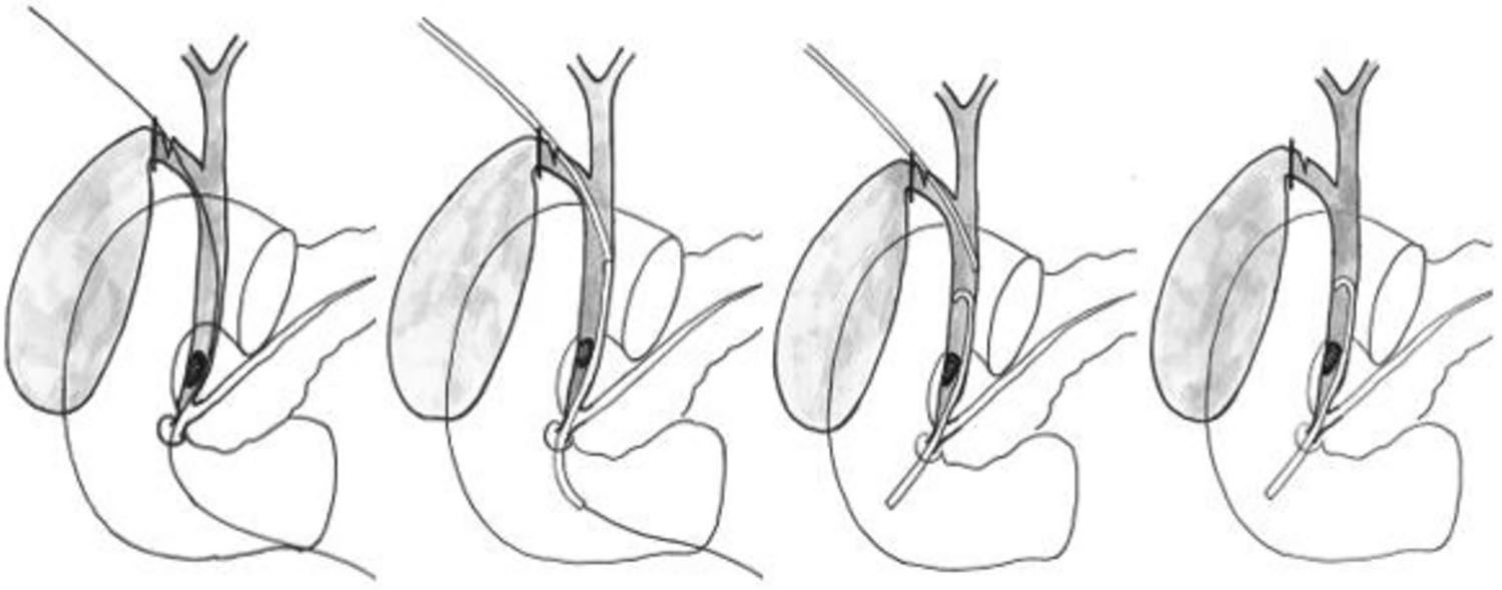
A guidewire is inserted in the cystic duct, and advanced into the duodenum via the common bile duct. The plastic stent is moved over the wire and pushed into the right position by a catheter. Once the stent passes the papilla, the wire is removed first, followed by the catheter. The procedure is performed under X-ray guidance

Antegrade placement of a stent in the common bile duct was first reported in 1998. Access was primarily achieved via choledochotomy by Gersin and Fanelli [7] and via the cystic duct by Chung [8]. The stent enables biliary drainage and pre-establishes biliary access for subsequent ERCP. Although never adequately evaluated, laparoscopic transcystic stenting has been used in clinical practice for decades and is listed as a treatment option for CBDS in the Society of American Gastrointestinal and Endoscopic Surgeons (SAGES) guidelines [9]. Publications from small case series report promising results [8, 10–15]. No study has, however, yet been published evaluating TCStent–ERCP against any alternative intraoperative treatment method. The aim of this study is to compare complication rates and outcomes of TCStent–ERCP and RV–ERCP strategies in the management of common bile duct stones detected during laparoscopic cholecystectomy.

## Materials and methods

The study was approved by the Swedish Ethical Review Authority in Stockholm, dnr 2023-05310-01 and dnr 2024-07105-02. Preregistration was conducted at ClinicalTrials.gov (NCT06817291). Reporting of this registry-based retrospective cohort study complies with the STROBE guidelines.

GallRiks, the Swedish Registry for Gallstone surgery and ERCP, is supported by the Swedish National Board of Health and Welfare. The registry has a national coverage of 94% for cholecystectomy and 86% for ERCP [6] and has been shown to have a high degree of accuracy when validated [16].

We extracted data from GallRiks on patients aged ≥ 18 years who underwent cholecystectomy at which CBDS were detected by IOC and managed by ERCP in the same session or later. One ERCP procedure in connection with the cholecystectomy was assessed for each patient. The cholecystectomies were performed between 1st January 2010 and 31st December 2023 at three hospitals conducting both TCStent–ERCP and RV–ERCP procedures during that period: Blekinge Hospital (Karlskrona); Sahlgrenska University Hospital (Gothenburg); and Ersta Hospital (Stockholm). The first two hospitals carry out acute and elective surgery while the third provides mainly elective surgery. The study size was determined by the number of eligible individuals available in the register without prior sample size calculation. Data was extracted on treatment strategy for CBDS, age, sex, the American Society of Anaesthesiologists (ASA) physical status classification, acute or elective cholecystectomy, additional procedures performed during the operation, procedure time for cholecystectomy and ERCP, difficult cannulation, and bile duct clearance at ERCP, length of stay, complications, unplanned readmission, and mortality. Data on Clavien–Dindo grading of complications was not possible to include since this was first introduced in GallRiks in 2021.

Acute cholecystectomy refers to surgery performed during a hospital stay with acute admission. Difficult cannulation in GallRiks is defined according to the European Society of Gastrointestinal Endoscopy (ESGE) guidelines as exceeding 5 min of cannulation attempts, 5 contacts with the papilla, or 2 unintended pancreatic duct cannulations [17]. Intra- and post-procedural complications within 30 days are recorded in GallRiks for both cholecystectomy and ERCP, resulting in four separate registrations for each patient in the study. If the ERCP is performed within 30 days after the cholecystectomy, only one 30-day follow-up is generated in GallRiks, and this follow-up registration is subsequently linked to both procedures.

We considered all complications in the same patient as one entity for each treatment strategy, as it cannot be strictly determined if the complication was associated with the cholecystectomy, the endobiliary stent, or the ERCP. Common bile duct obstruction, requiring unplanned intervention with ERCP or reoperation, identified at the 30-day follow-up, is classified as a complication in GallRiks. If multiple complications were recorded for the same patient, only the most severe was considered. In this study, we classified any complication directly attributable to surgery or ERCP as a surgical complication, including post-procedural pancreatitis. Between 2010 and 2020, placement of a transcystic stent or a guidewire was registered as the same variable in GallRiks. To determine which of these methods had been used, the operation notes in the medical records were reviewed at each hospital.

Primary outcome was complications within 30 days including overall complications, surgical complications, and post-procedural pancreatitis.

Secondary outcomes included procedure time, difficult cannulation during ERCP, bile duct clearance at ERCP, length of stay, unplanned readmission, and mortality within 30 days.

Statistical analyses were conducted in R, version 4.4.1. Differences in baseline characteristics between the groups were calculated by Chi-squared test, except for age where Mann–Whitney *U* test was applied. Comparison of groups regarding complications was conducted using multivariable logistic regression analyses adjusted for age, sex, ASA class, and acute or elective surgery. ASA class was dichotomised into ASA class 1–2 and 3–4 in the analyses. Procedure time for ERCP and length of stay were analysed by Mann–Whitney *U* test. Bile duct clearance (complete, incomplete, or no stone extraction) was analysed with Fisher’s exact test. Difficult cannulation and unplanned readmission were assessed with Chi-squared test. A *p*-value < 0.05 indicated statistical significance.

Two post hoc analyses were conducted. First, a subgroup analysis was performed among patients undergoing elective surgery using logistic regression adjusted for age, sex, and ASA classification. Second, propensity score matching was performed because the proportion of elective versus acute surgery differed substantially between groups. We used 1:2 nearest-neighbor matching without replacement, with a caliper of 0.2 standard deviations of the logit of the propensity score, based on age, sex, ASA class, and elective or acute surgery, followed by logistic regression analysis with standard errors clustered at the matched set level.

Statistical analyses were reviewed and verified by an independent statistician.

## Results

**Fig. 2:**
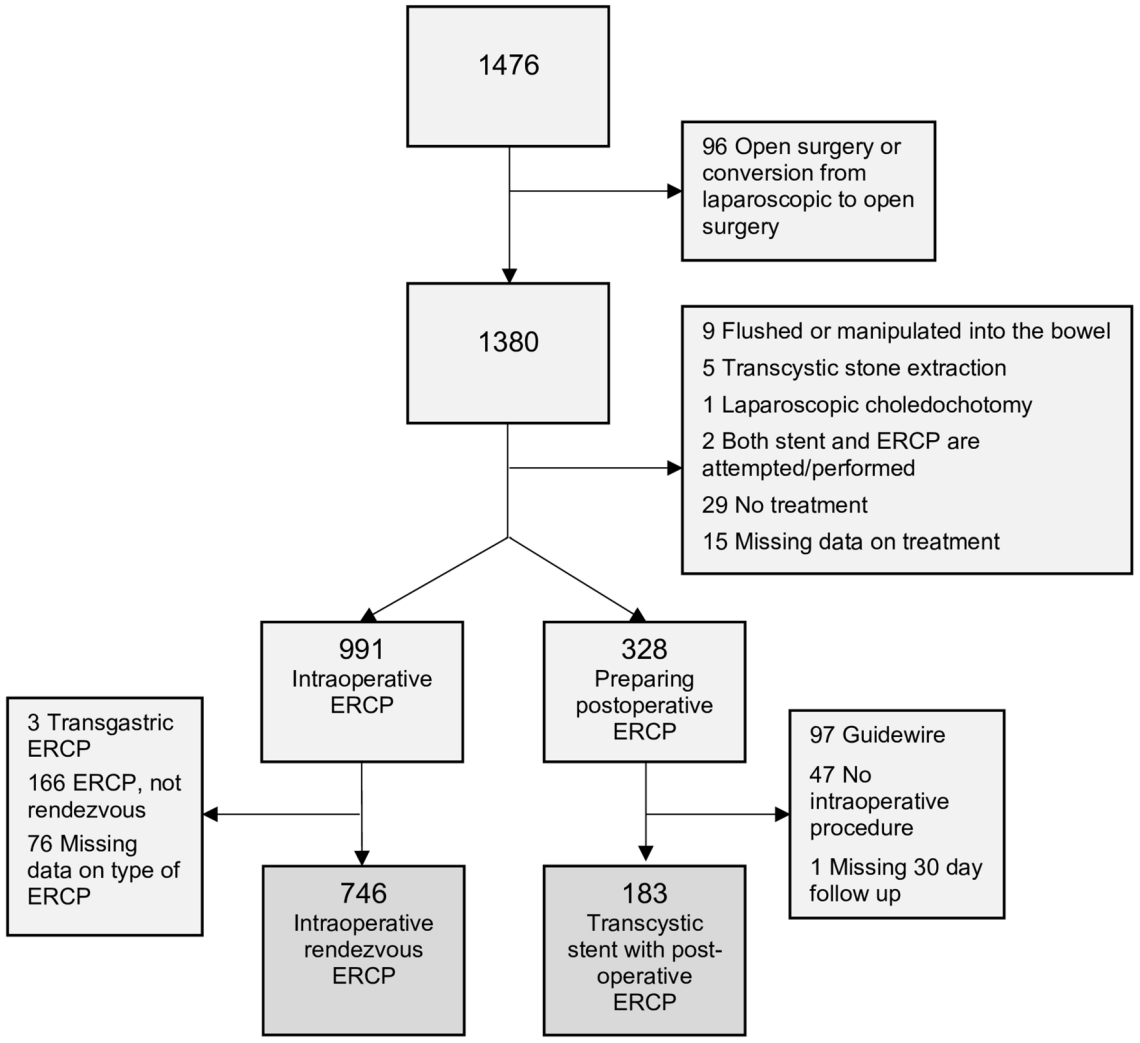
Cholecystectomies in patients ≥ 18 years at Blekinge Hospital, Sahlgrenska University Hospital and Ersta Hospital with CBDS encountered intraoperatively followed by ERCP registered in GallRiks between 1st January 2010 and 31st December 2023

During the study period, the three hospitals registered 1476 cholecystectomies, in which CBDS were encountered at IOC and ERCP was performed simultaneously or later. Patients managed with other techniques for intraoperative ERCP than rendezvous, or who were prepared for postoperative ERCP without a transcystic stent, were excluded, as were patients undergoing open surgery or transgastric ERCP. In cases where both stent placement as well as intraoperative ERCP were attempted, it was not possible to distinguish the effect of either technique, therefore, these patients were excluded. One patient in the TCStent–ERCP group, with missing data at the 30-day follow-up, was also excluded. Following these criteria, 929 eligible patients were identified: 183 underwent transcystic stenting with postoperative ERCP and 746 were treated with intraoperative rendezvous ERCP (Fig. 2).

Distribution of ASA classification did not differ between groups (Table 1). In the TCStent–ERCP group, the median age was 5 years higher, the proportion of men lower (*p* = 0.01), and the proportion of acute procedures lower (*p* < 0.001) compared to the RV–ERCP group (Table 1). The percentage of patients with cholecystitis among the acutely operated was similar in the groups (Table 1).

**Table 1:**
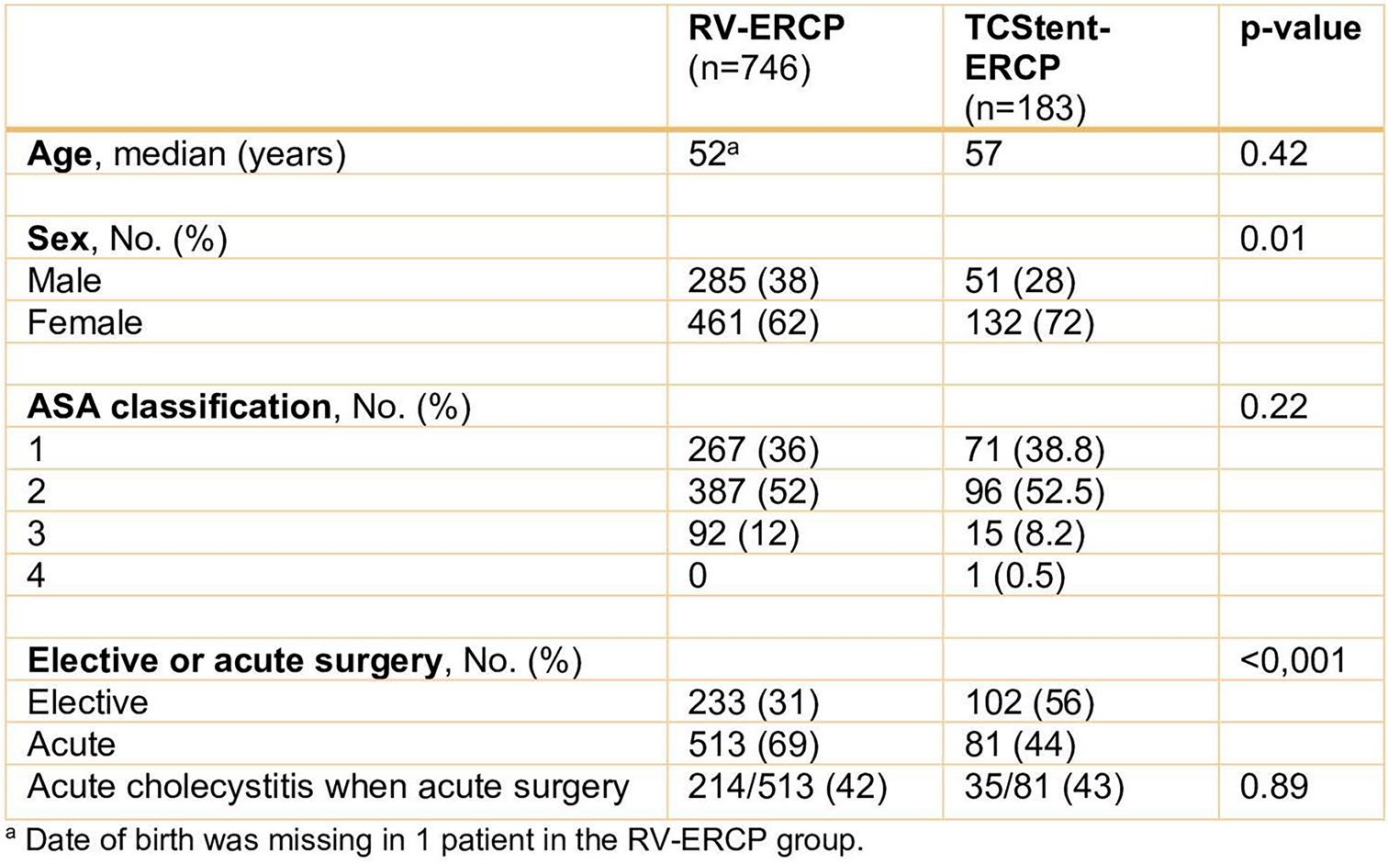
Baseline characteristics at cholecystectomy

The proportion of patients with overall complications, surgical complications, and post-procedural pancreatitis were 11%, 10%, and 5.5% in the TCStent–ERCP group and 19%, 14% and 3.9% in the RV–ERCP group (Table 2). In multivariable logistic regression analysis, TCStent–ERCP was associated with significantly lower odds of overall complications compared with RV–ERCP (OR 0.60, 95% CI 0.35–0.97). However, no differences were observed between the groups regarding surgical complications or post-procedural pancreatitis (Table 2). All patients registered with the complication ‘bile duct obstruction’ at the 30-day follow-up were treated with unplanned ERCP. Complications classified as non-surgical included conditions such as thrombosis, pneumonia, and urinary retention necessitating bladder catheterization.

**Table 2:**
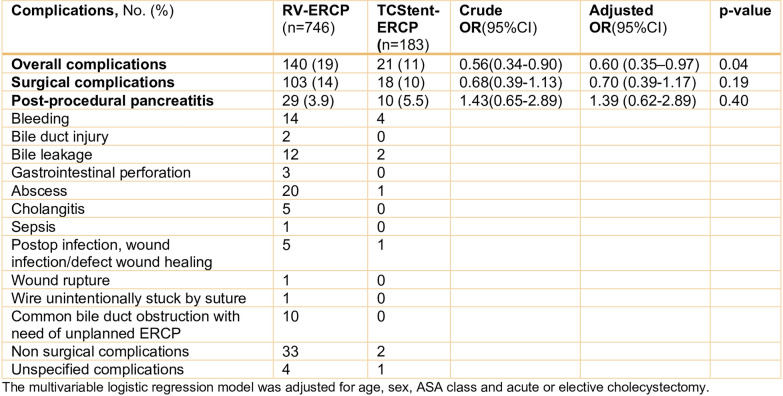
Primary outcomes, complications from cholecystectomy, and ERCP

No co-planned surgical procedure that could be assumed to have influenced the outcome was performed during any cholecystectomy.

The procedure time for the cholecystectomy itself was not analyzed separately, since RV–ERCP was sometimes included in the time for cholecystectomy and sometimes not. In the TCStent–ERCP group, the median procedure time for ERCP was shorter (*p* < 0.001), whereas difficult cannulation was more common (*p* < 0.001) compared to the RV–ERCP group (Table 3). A high rate of bile duct clearance, evaluated immediately after ERCP, was achieved with both methods, with no difference between the groups (Table 3).

**Table 3:**
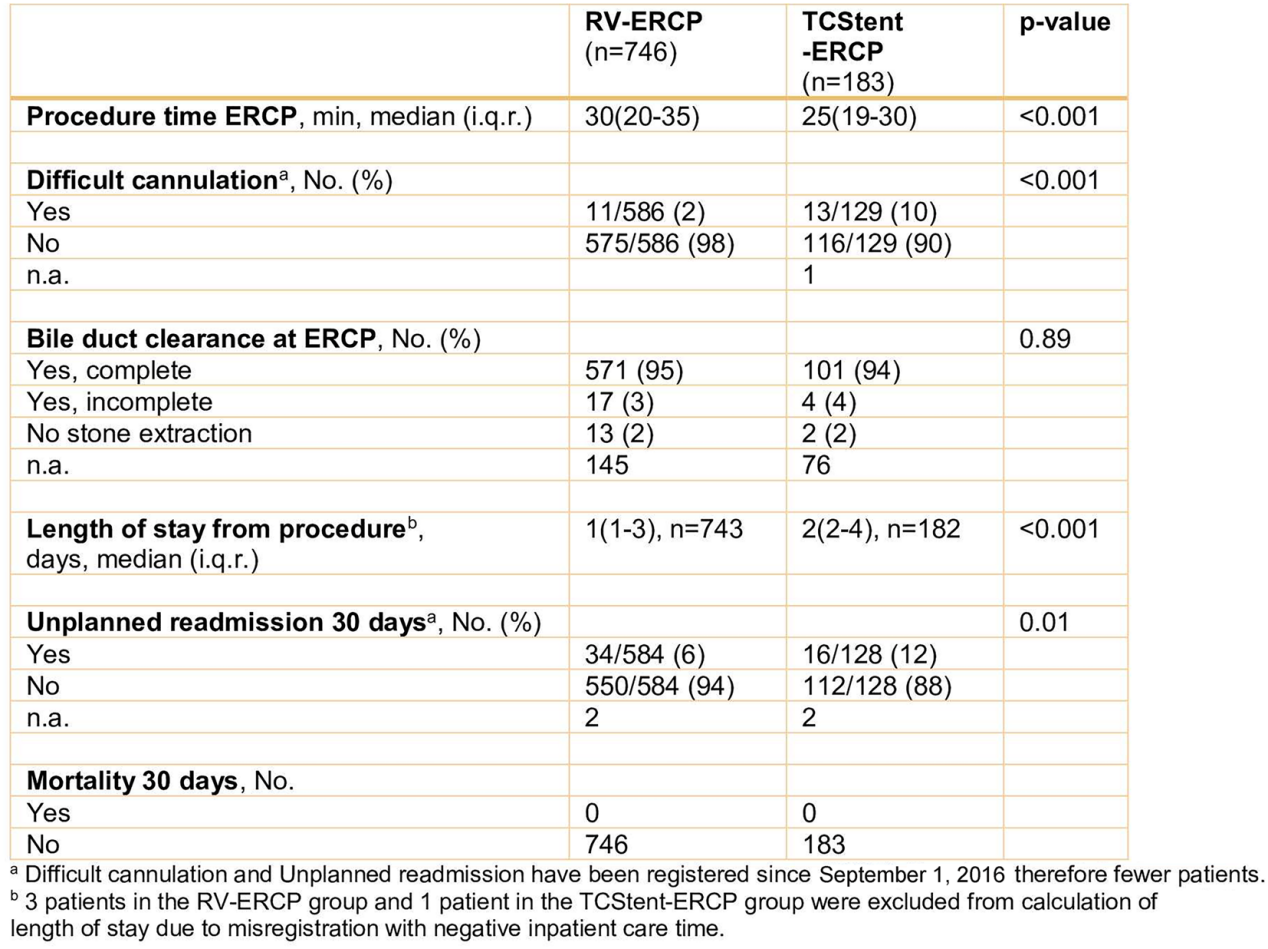
Secondary outcomes

Date of admission was missing for 90% of the patients and therefore, time from cholecystectomy until discharge was used as a proxy for length of stay. Likewise, in cases with two admission periods, the time from ERCP to discharge was used. Median total length of stay was one day longer in the TCStent–ERCP group compared with the RV–ERCP group (*p* < 0.001) (Table 3). In the TCStent–ERCP group, subsequent ERCP was performed during the same admission as the cholecystectomy in 15% of cases, on an outpatient basis in 34%, and during a separate inpatient admission in 51%. Using the TCStent–ERCP method, the median interval between cholecystectomy and ERCP was 22 days, ranging from the primary admission to weeks or months later.

Unplanned readmission within 30 days, regardless of indication, was more common with TCStent–ERCP than with RV–ERCP (*p* = 0.01) (Table 3).

There was no 30-day mortality recorded in either of the groups (Table 3).

In a post hoc analysis of elective procedures (*n* = 335), after excluding those who underwent cholecystectomy in an acute care setting, TCStent–ERCP was associated with lower odds of overall complications compared with RV–ERCP; however, this difference did not reach statistical significance (OR 0.51, 95% CI 0.23–1.03).

A post hoc analysis was also conducted after propensity score matching, which achieved good covariate balance, with all standardized mean differences < 0.1. In the matched cohort, patients treated with TCStent–ERCP (*n* = 183) had significantly lower odds of overall complications compared with those treated with RV–ERCP (*n* = 349) (OR 0.54, 95% CI 0.32–0.91).

## Discussion

In this retrospective GallRiks registry-based study, the TCStent–ERCP method was associated with lower overall complication rates but with no differences in surgical complications or post-procedural pancreatitis compared with the RV–ERCP method. In addition, ERCP procedure time was slightly shorter whereas total length of stay was longer with TCStent–ERCP. Bile duct clearance was similarly high with both techniques. Taken together, these findings demonstrate that TCStent–ERCP is a safe and efficacious alternative to RV–ERCP for management of CBDS encountered at IOC.

The cause of the difference in overall complication rates is not entirely clear. An unplanned intraoperative ERCP will likely increase the surgical stress the patient is exposed to, potentially increasing the risk of complications compared with ERCP performed in a later elective setting. Although adjusted for, it remains questionable whether the higher number of acute cholecystectomies in the RV–ERCP group may have contributed to the increased overall complication rates. It should be noted that the percentage of patients with cholecystitis among acute cholecystectomies did not differ between groups. As in the overall cohort, the post hoc subgroup analysis of elective procedures, also showed lower odds of complications with TCStent–ERCP compared with RV–ERCP, although not statistically significant. Post hoc logistic regression analysis after propensity score matching yielded results consistent with the adjusted multivariable model, demonstrating significantly fewer overall complications with the TCStent–ERCP method. This indicates that differences in proportions of acute procedures between the groups cannot alone explain the observed results.

Procedure time for ERCP was shorter in the TCStent–ERCP group, but length of stay after the procedure was longer compared with the RV–ERCP group. The ERCP after transcystic stenting is always performed in a prearranged elective setting, which could be assumed to be associated with a shorter procedure time. A consequence of the TCStent–ERCP strategy is that it requires two episodes of anesthesia and often two separate hospital admissions, apparently contributing to the overall longer hospital stay in this group. The higher rate of unplanned readmission in the TCStent–ERCP group may be attributed to symptoms related to remaining CBDS or to the stent itself. However, the higher rate of readmissions was not reflected by a higher risk of complications.

Since the procedure time for cholecystectomy could not be accurately assessed, we were not able to estimate how much placement of the stent prolonged the procedure. However, it has been reported that insertion of a transcystic stent adds approximately 15 min to the procedure [8, 10–13] and causes no or few complications [8, 11, 12, 15]. Furthermore, it has been repeatedly reported that transcystic stenting could facilitate subsequent ERCP [10, 11, 13, 15]. In the present study, TCStent–ERCP was, if anything, associated with lower risk of complications compared to RV–ERCP, and with similarly high rates of bile duct clearance.

The incidence of PEP was 5.5% following TCStent–ERCP and 3.9% following RV–ERCP in our study, with no statistically significant difference between the groups. Previously reported incidence rates of PEP range from 3.5% to 9.7% [18–21], which corroborates our findings.

Previous reports on the TCStent–ERCP method are limited to single-center studies with considerably smaller cohorts [8, 10–15]. A major strength of our study is therefore that it represents by far, the largest cohort so far evaluating the TCStent–ERCP method, and it is the first to compare TCStent–ERCP with another intraoperative treatment approach. This provides valuable insights into the effectiveness and safety of the intervention. In addition, our study considers complications from both the cholecystectomy and ERCP, which allows the entire process to be assessed. Importantly, study data are based on GallRiks, a well-validated register with a rigorous 30-day follow-up, and the use of a transcystic stent was verified against medical records to ensure accuracy in cases with any ambiguous data in GallRiks.

Our study is not without weaknesses. As data were restricted to three hospitals and the results are primarily applicable to settings where IOC is performed during cholecystectomy, the generalisability of our findings may be reduced. It is possible that the surgeon opted for transcystic stenting when ERCP expertise was not available for rendezvous ERCP. Selection bias can therefore not be ruled out. The inability to report the total length of stay from admission might be considered a limitation. On the other hand, excluding the varying time from admission to procedure might be an advantage, since this includes the waiting time commonly encountered in acute surgery. Therefore, the reported length of stay in the study might reflect the postoperative course better. Comparison with other treatment options for management of CBDS, such as choledochotomy or transcystic stone extraction by choledochoscopy, was unfortunately not possible in our study.

ERCP requires considerable training. Placing a transcystic stent laparoscopically, however, could likely be performed with a short learning curve for a surgeon experienced in gallbladder surgery, enabling relatively rapid implementation. A considerable part of cholecystectomies today is performed in units without or with limited access to ERCP. Relocation of gallbladder surgery from hospitals with acute admission to other units has raised the question of how to provide a safe alternative to RV–ERCP. Swedish guidelines require an established strategy for managing common bile duct stones intraoperatively if such suspicion exists preoperatively [22]. With the TCStent–ERCP method available, treatment of CBDS can consistently be provided.

In summary, if CBDS are diagnosed at IOC during laparoscopic cholecystectomy, our data demonstrates that TCStent–ERCP is a safe, effective, and readily available alternative to RV–ERCP. This may be particularly valuable in surgical units with limited or lacking resources to perform unplanned ERCP.
